# Supplementary material for: scAAV2-Mediated Expression of Thioredoxin 2 and C3 Transferase Prevents Retinal Ganglion Cell Death and Lowers Intraocular Pressure in a Mouse Model of Glaucoma
Source: Int J Mol Sci. 2023 Nov 13;24(22):16253. doi: 10.3390/ijms242216253 (PMC10671512; doi:10.3390/ijms242216253)
Supplement: Supplementary file 1 [file ijms-24-16253-s001.zip › ijms-2640204-supplementary.pdf]

Supplementary Materials

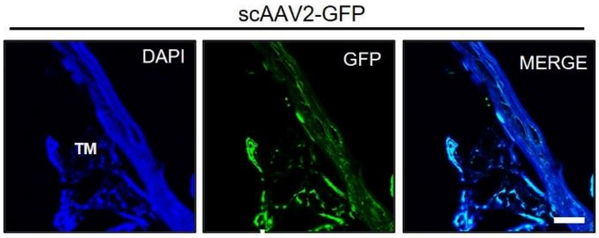

Figure s1. Expression of reporter transgene after single intracameral injection of scAAV2-GFP. Delivery of the reporter gene encoding a green fluorescent protein (GFP) to the anterior segment of the living mouse after a single injection of the viral vector. Scale bar, 50 μM.
